# Identification of Deformation Effects While Shaping the Material Surface Relief Due to Burnishing Treatment

**DOI:** 10.3390/ma17225635

**Published:** 2024-11-18

**Authors:** Andrzej Zaborski, Robert Rogólski, Stanisław Grzywiński

**Affiliations:** 1 Faculty of Mechanical Engineering, Częstochowa University of Technology, ul. J.H. Dąbrowskiego 69, 42-201 Częstochowa, Poland; andrzej.zaborski@pcz.pl; 2 Faculty of Mechatronics, Armament and Aerospace, Military University of Technology, ul. gen. Sylwestra Kaliskiego 2, 00-908 Warsaw, Poland; stanislaw.grzywinski@wat.edu.pl

**Keywords:** burnishing, deformation zone formation, metal surface treatment

## Abstract

This study analyses a set of phenomena occurring in the burnished surface layer at the initial moment of deformation formation. The aim of the present research was to explain the phenomena occurring in the top layer of the material during burnishing. The presented analyses include selected laboratory and experimental studies of the process involved in forming burnished surface layers. As shown, conducting an analysis of these processes is purposeful and important because the processes affecting final deformations determine the definitive properties of the burnished surface layers. The final results should help to increase the durability and smoothness of the surface of the products obtained. The feasibility of applying computer technology to determine the three-dimensional shape of the deformation zone formation based on measurements of the stereometry of the contact zone of the burnishing tool with the workpiece material is presented. The process of forming a deformation zone was analysed, revealing that irregularities left over from prior treatment are permanently deformed, and a new structure of irregularities is formed on the machined surface, conditioned by the mechanical, geometric, and kinematic factors of the process. Crucial to this are qualities such as the burnishing load (pressure), the type, shape, and dimensions of the tool, the properties of the workpiece material, and the roughness of the surface before burnishing. The analyses presented here include the first stage of processing, in which initial contact is made with the workpiece, and the period of actual processing, during which plastic deformation of the material occurs in three perpendicular directions, leading to the formation of a material wave on the machined surface just in front of the burnishing tool.

## 1. Introduction

One of the commonly used finishing methods to improve the functional (operational) properties of machine parts is surface burnishing. This process uses tools such as a ball, disc, roller, or a similar shape to create a localized plastic deformation in the surface layer of an object by direct contact between the tool tip and the machined surface. The processes occurring within the deformation zone, analyzed in the study, determine the final properties of the surface layers subjected to burnishing treatment. The deformation process can lead to the immunization of the surface layer of the object to the effects of mechanical, thermal, and chemical factors occurring during operation. The immunization of the surface layer of the processed object occurs thanks to the creation of a new, advantageous structure with increased microhardness and thanks to the advantageous distribution of residual stresses. Burnishing allows for a relatively simple way to obtain high surface smoothness and improve the physicomechanical properties of the surface layer, aimed at, among others, increasing fatigue strength and resistance to wear in friction conditions.

The available literature devotes little attention to the processes occurring within the formation of the deformation zone during burnishing. This does not seem to be fully justified because it is the processes occurring within this zone that determine the final properties of the burnished surface layer. In professional literature, contact surfaces are most often determined in an approximate manner due to difficulties in the analytical or experimental determination of the actual contact zone of the burnishing element with the machined surface. Meanwhile, the actual contact surfaces, due to their dependence on the height of the material wave formed in front of the burnishing element, have a complex spatial shape. Hence, from the point of view of the proper selection of technological parameters of the burnishing process, it is important to determine the actual shapes and size of the formed deformation zone. This would enable determining the actual value of contact stresses occurring during burnishing processing. Conducting research aimed at describing the phenomena occurring during burnishing at the formation of the contact zone is therefore fully justified.

**Figure 1:**
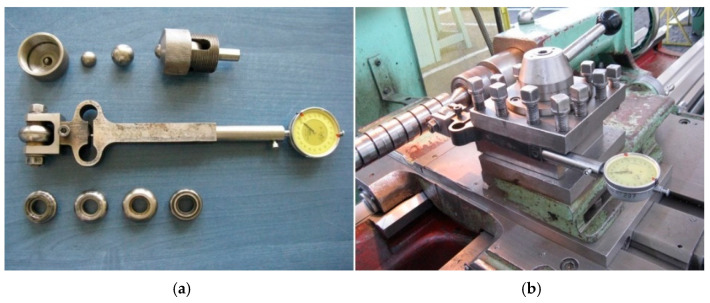
Tools used for burnishing: (**a**) tool in details; (**b**) tool position during machining.

Comprehensive analyses of the surface layer formation processes obtained by various burnishing methods have shown that the method and processing parameters adopted for the machining process determine the final performance of the as-machined surface. Burnishing enables relatively easy control of the surface layer properties produced [1,2]. Experimentally verified theoretical analysis has shown that processing with apparently similar processing parameters can cause changes in the deformation pattern, resulting from a change in the kinematic system of the tool’s interaction with the workpiece, which can lead to surface layers with a significantly different performance [3,4]. The study presented here concerns the feasibility of using modern techniques for visualizing surface stereometry to analyse changes in surface relief that occur as a result of shaping the tool contact zone with the workpiece material during surface treatment by burnishing. The analyses include the first stage of processing, in which initial contact is made with the workpiece, and the period of actual processing, during which deformation of the material occurs in three perpendicular directions, leading to the formation of a wave of material on the machined surface in front of the burnishing tool. Surface treatment by burnishing is performed to achieve two basic processing objectives: strengthening the material at the depth of the surface layer and improving the stereometric condition of the processed surface. The objective of this study was to establish a relationship between the processing parameters of burnishing with the course and effects of the process of forming the burnished surface layer. This study was carried out maintaining the typical parameters used in ball-and-disc stress relief burnishing of C55 steel as the burnished workpiece. By using differently shaped tools (Figure 1) for burnishing under the same processing conditions, or by varying the processing parameters, it is possible to achieve different degrees of the deformation of irregularities [5,6]. Depending on the requirements, it is possible to achieve partial deformation of irregularities when the burnishing tool has too large a profile radius and cannot generate the specific pressures necessary to fully deform the irregularities (the valleys of the notches in the irregularities remain on the surface after burnishing), as well as their final deformation when the burnishing tool pushes a high-pressure load on the burnishing area that any irregularities are totally deformed.

In the scientific literature on the surface treatment of metallic materials, there are many interesting studies investigating the effects of burnishing treatment on material quality or its outer microgeometry. Ball or roller burnishing affects technological quality [7], integrity [8,9], and tribological properties of the outer area of the treated material [10]. Forming the topometric profile of the material surface, shaping its textures, and obtaining roughness characteristics are described in [11,12,13]. The publications cited here present exemplary studies of selected steel alloys subjected to surface burnishing. Plastic deformation of the top layer of the material is to improve mechanical properties, including surface hardening, increased fatigue strength, improved resistance to wear and corrosion, and improved adaptation in cooperating tribological connections. The studies presented herein serve to demonstrate the method and present the results, the usefulness of which may prove significant only in the context of selecting a technology dedicated to a specific manufacturing process.

The intention of the undertaken study was to enable controlled forming the burnished surface layers by selecting and adjusting the processing parameters that govern the deformation image. This would enable the burnishing process to be operated in such a way as to effectively achieve the desired performance of the machined surfaces. By analyzing the behaviour of the material in the deformation forming zone, it becomes possible to specify the optimal parameters of the material surface after processing.

The main purpose of the conducted analyses was to provide a visual image of the deformed surface and identification of the deformation profile in two perpendicular directions. Target control of the deformation profile and the possibility of adjusting the dimensions of the profile curve introduces the perspective of quality control of the machined surface as a function of burnishing parameters and surface stereometry parameters. As a consequence of finding close connections between geometric parameters and physical properties of the surface layer, it will probably be possible to develop functional relationships ensuring active control of the mechanical properties of the machined layer.

## 2. Analyses of the Stereometry Constitution in the Deformation Zone—Cases of Burnishing Treatment

### 2.1. Initial Stage of the Deformation Formation Process

**Figure 2:**
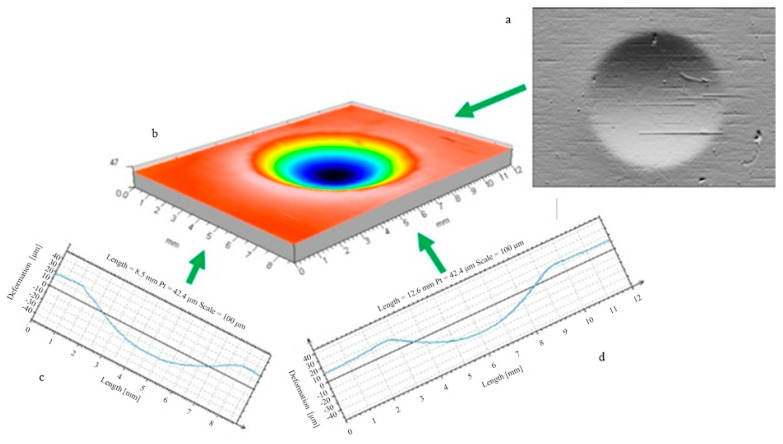
Visualisation of the material surface deformed by the burnishing tool (ball indenter) and the burnished surface; radius R = 12.5 mm, burnishing force F = 5 kN. ((**a**) top image, (**b**) stereometric image, (**c**,**d**) 2D deformation profiles in the lateral and longitudinal direction).

The burnishing process must begin with an initial contact between the burnishing tool and the workpiece surface. This first moment of the deformation formation process involves plunging the tool (ball or disc) into the workpiece material. This study analysed the processes of forming the stereometry of the indentation area of a tool (indenter) into a flat material. The stereometric measurements were completed with a computerised measuring station, the New Form Talysurf 2D/3D 120 profilographometer. It was possible to record and analyse the actual 3D shape of the area of the deformation zone formed by the indenting tool. An example of the shape of the deformation zone is shown in Figure 2.

Of course, it is important to appreciate that the analysed processes of the indentation of the tool into the flat material only reflect, to a certain approximation, the set of phenomena occurring during the initial moment of the burnishing process. However, the conclusions that can be drawn from them allow for a better understanding of the processes occurring in the contact zone of the tool with the workpiece material during actual burnishing. To fully understand the phenomena occurring in the contact area between the tool and the workpiece, it is necessary to carry out analyses that take into account the actual shape of the contact zone and the full kinematics of the process.

The performance of the processes occurring in the initial stage of the deformation process during burnishing was carried out on a test bench, which facilitated the burnishing process for cylindrical surfaces (shown in Figure 1) to be completed using burnishing tools similar to those used in real-world production processes. This study involved detailed analyses of the formation processes of the stereometry of the tool’s indentation area in the material during the successive stages of the deformation focus formation process.

**Figure 3:**
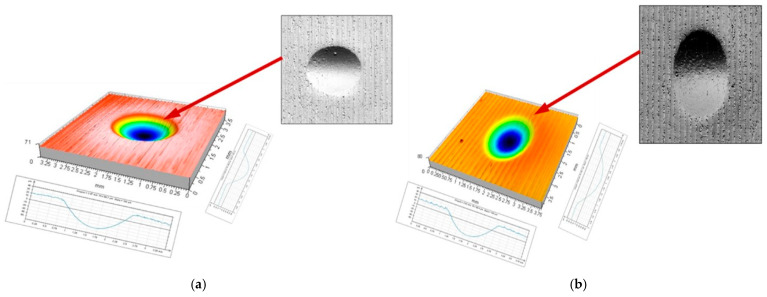
Beginning of the deformation formation process—tool penetration into the workpiece surface: (**a**) ball indenter (R = 5 mm, F = 2.75 kN); (**b**) disc indenter (R_k_ = 5 mm, F = 2.75 kN).

**Figure 4:**
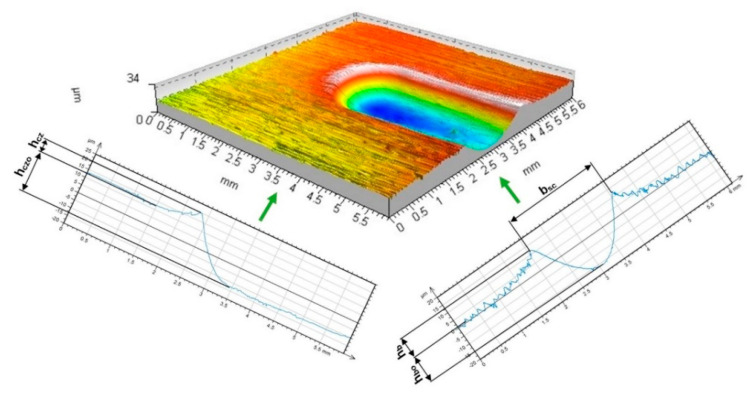
Forming the recess groove during the burnishing tool motion—ball indenter (R = 13.5 mm, F = 2.75 kN).

The indentation process of the burnishing tool, initiated by contact between the tool and the workpiece surface, continues until the final depth is reached under the action of the increasing indentation force (pressure). The ball indentation shown in Figure 3a had a diameter of 1.87 mm and a depth of 73 μm, and the disc indentation shown in Figure 3b had an elliptical shape with axis lengths of 1.84 and 2.55 mm and a depth of 59 μm. A continued burnishing process requires the emergence of forces moving the tool in a direction tangential to the workpiece surface. The movement in the circumferential direction (the direction of the main movement) of the cylindrical surface to be burnished is the most important in this case. It is critical for the formation of the local accumulation of plasticized material ahead of the burnishing tool. The most important geometrical parameters defining the geometrical characteristics of the deformation focus are summarized in Figure 4. As soon as the burnishing tool leaves the static indentation zone, the material begins to be deformed in the circumferential direction. Both frontal and lateral waves of deformed material emerge, forming the path of the tool movement (Figure 4—b_sc_ = 2.02 mm, h_b_ = 8.6 μm, h_bo_ = 18.5 μm, h_cz_ = 9.8 μm, and h_czo_ = 19.1 μm).

**Figure 5:**
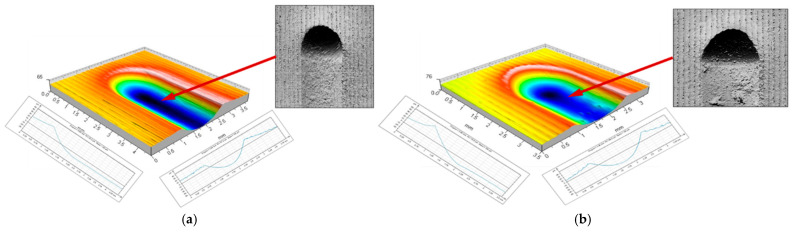
Start of burnishing tool movement: (**a**) ball indenter (R = 5 mm, F = 2.75 kN); (**b**) disc indenter (R_k_ = 5 mm, F = 2.75 kN).

As a result of this process, a groove-like mark formed behind the burnishing tool, and a wave of accumulated material appears before the moving tool. The deformation parameters in Figure 5a are: b_sc_ = 1.74 mm, h_b_ = 15.3 μm, h_bo_ = 39.2 μm, h_cz_ = 25.5 μm, and h_czo_ = 46.4 μm; deformation parameters in Figure 5b—b_sc_ = 1.6 mm, h_b_ = 4.8 μm, h_bo_ = 41.2 μm, h_cz_ = 10.5 μm, and h_czo_ = 41.5 μm.

During the initial period of displacement of the tool, which causes deformation, the wave of material forming in front of the tool had a height comparable to that of the wave of material formed around the tool during the plunge. The deformation resistance in the direction of the main movement being performed was initially insignificant. The deformed material began to form waves shaped on either side of the burnishing path being formed. There was compression and displacement of material in the frontal contact zone, resulting in a marked increase in the height of the contact zone. The material underwent secondary displacement within the previously deformed layer. There was a clear increase in the main force accompanying the burnishing process in progress. As the analyses revealed, the material of the surface layer of the deformation zone underwent significant strengthening depending on the processing parameters used. The type and geometry of the burnishing tool and the applied burnishing force proved to be the most important.

**Figure 6:**
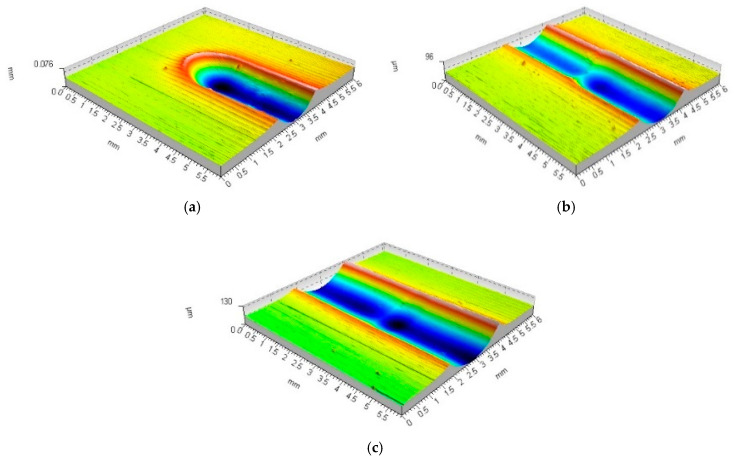
Forming the recess groove during the burnishing tool motion—ball indenter (R = 5 mm, F = 2.75 kN): (**a**) start of movement (b_sc_ = 1.74 mm, h_b_ = 15.3 μm, h_bo_ = 39.2 μm, h_cz_ = 25.5 μm, h_czo_ = 46.4 μm); (**b**) third tool pass (b_sc_ = 1.89 mm, h_b_ = 22.0 μm, h_bo_ = 36.1 μm, h_cz_ = 12.3 μm, h_czo_ = 9.5 μm); (**c**) tenth tool pass (b_sc_ = 2.14 mm, h_b_ = 29.3 μm, h_bo_ = 47.6 μm, h_cz_ = 9.5 μm, h_czo_ = 6.9 μm).

**Figure 7:**
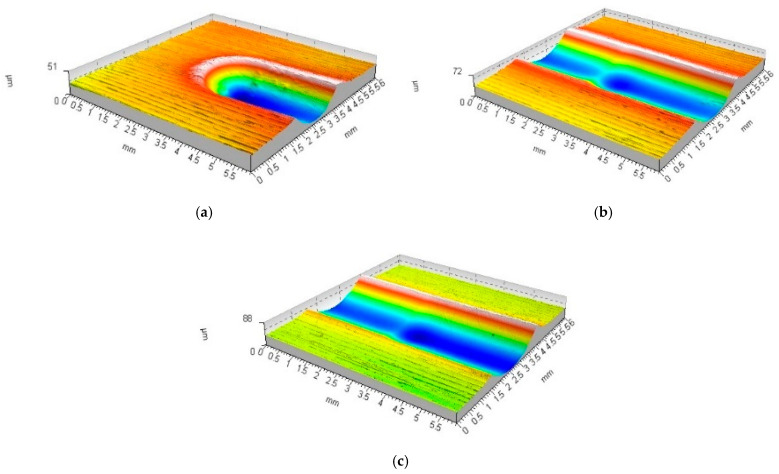
Forming the recess groove during the burnishing tool motion—ball indenter (R = 9.5 mm, F = 2.75 kN): (**a**) start of movement (b_sc_ = 2.06 mm, h_b_ = 4.8 μm, h_bo_ = 38.7 μm, h_cz_ = 12,8 μm, h_czo_ = 35.2 μm); (**b**) third tool pass (b_sc_ = 2.34 mm, h_b_ = 8.7 μm, h_bo_ = 37.8 μm, h_cz_ = 5.7 μm, h_czo_ = 7.3 μm); (**c**) tenth tool pass (b_sc_ = 2.46 mm, h_b_ = 11.5 μm, h_bo_ = 39.5 μm, h_cz_ = 8.6 μm, h_czo_ = 2.2 μm).

Analysis of the rolling ball burnishing process carried out allowed the conclusion that the process would achieve stability. In the first phase, the tool was plunged to a process-specific depth; in the second stage, the process-specific wave height in front of the burnishing tool was formed; and in the third stage, the final height of both waves formed along the emerging tool path was reached. Once these processes are complete, the parameters of the formed deformation are stabilised. There was full deformation of the material both in front of the tool and on both sides of the formed tool path (Figure 6a and Figure 7a). Compared to the first moment of deformation emergence, the distribution of forces with which the tool interacted with the workpiece material changed. This stage of machining could continue until—after a full rotation of the workpiece—the tool hit the mark made in the previous pass. If the burnishing is carried out without the application of the feed rate characteristic of the actual burnishing process, an annular groove appears on the surface of the material. The tool started the next pass by entering the previously deformed material (Figure 6b and Figure 7b). During this movement, the previously formed groove was deepened and widened.

The entry of the tool into a previously deformed area (closing of the tool path being created) led to a change in the conditions for the formation of the deformation. The wave of material accumulated ahead of the tool disappeared. At the same time, both waves formed along the tool’s path of movement were clearly deformed. Each successive pass of the burnishing tool resulted in increasing deformation and strengthening of the material. The analyses allowed the authors to conclude that the tool penetration depth increment becomes smaller with each successive pass. The depth of the formed tool path stabilised after about 10–20 passes of the burnishing tool (Figure 6c and Figure 7c). A further burnishing process hardly led to any further increase in deformation.

**Figure 8:**
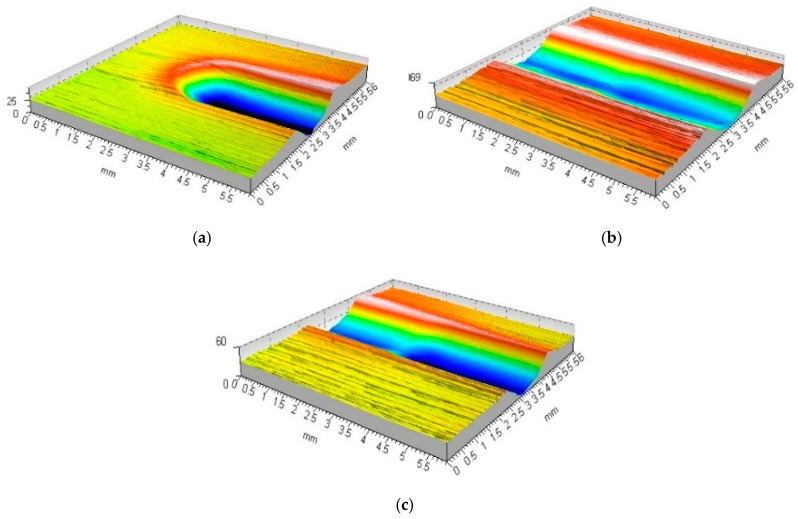
Forming the recess groove during the burnishing tool motion—disc indenter (R_k_ = 10 mm, F = 2.75 kN): (**a**) start of movement (b_sc_ = 1.23 mm, h_b_ = 3.7 μm, h_bo_ = 36.3 μm, h_cz_ = 9.4 μm, h_czo_ = 29.6 μm); (**b**) third tool pass (b_sc_ = 2.18 mm, h_b_ = 5.4 μm, h_bo_ = 32.2 μm, h_cz_ = 6.1 μm, h_czo_ = 3.3 μm); (**c**) tenth tool pass (b_sc_ = 2.45 mm, h_b_ = 11.6 μm, h_bo_ = 39.8 μm, h_cz_ = 1.8 μm, h_czo_ = 0.9 μm).

**Figure 9:**
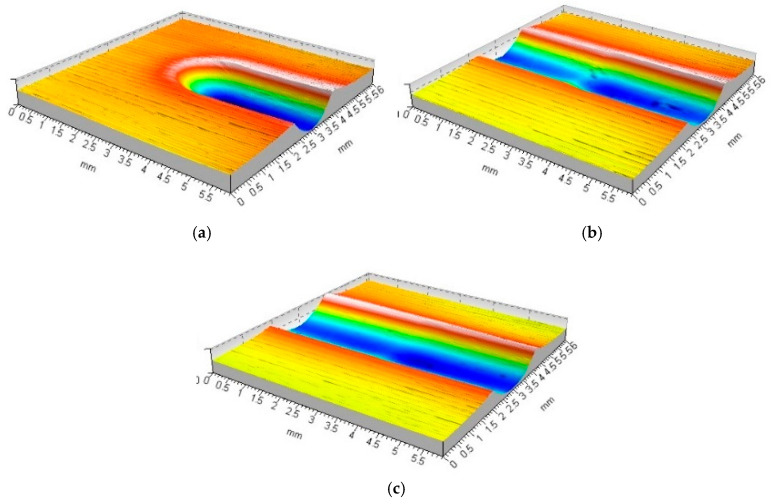
Forming the recess groove during the burnishing tool motion—disc indenter (R_k_ = 5 mm, F = 2.75 kN): (**a**) start of movement (b_sc_ = 1.61 mm, h_b_ = 3.8 μm, h_bo_ = 42.2 μm, h_cz_ = 10.5 μm, h_czo_ = 41.5 μm); (**b**) third tool pass (b_sc_ = 1.94 mm, h_b_ = 10.1 μm, h_bo_ = 51.2 μm, h_cz_ = 6.1 μm, h_czo_ = 8.5 μm); (**c**) tenth tool pass (b_sc_ = 2.31 mm, h_b_ = 17.7 μm, h_bo_ = 68.8 μm, h_cz_ = 4.5 μm, h_czo_ = 2.6 μm).

An analysis of the rolling disc burnishing process revealed that it progressed in a similar manner to the already discussed ball burnishing process. In Figure 8 and Figure 9, however, differences are noticeable due to the change in geometry of the tool producing the deformed zone. The significantly larger contact area between the tool and the burnished area resulted in a reduction of the specific contact stresses emerging in the process. The process stabilised much more quickly, and just a few successive passes of the burnishing tool led to stabilisation of the process and a practical halt to the increasing deformation.

Analysis of the processes accompanying the burnishing carried out by the tool movement in the axial direction (the burnishing feed) allowed an approximation to be made of the processes occurring in the initial period of roughness formation during burnishing. It becomes important here to be able to trace the geometrical and kinematic characteristics of the set of phenomena occurring in the area of the deformation focus [14]. Under real-life machining conditions, similar processes occur when processing with very low feed rates. The analysis of deformations present in both the circumferential and axial directions of the deformed cylindrical surface is important here.

### 2.2. Forming the Geometrical Structure of the Deformation Zone

**Figure 10:**
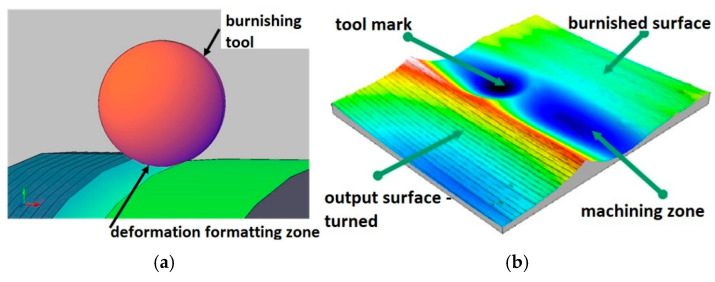
(**a**) Image of the machining process; (**b**) development of the contact area between the tool and deformable surface.

For the sake of the study presented here, the most relevant was the analysis of the actual shape of the deformation zone, considering the kinematics of the process and the stereometry of the surface after burnishing. Consideration was given to the feasibility of using a modern technique of visualizing surface stereometry to analyse the phenomena occurring during the formation of the tool’s contact zone with the workpiece material during surface treatment by burnishing. During the analyses, rolling burnishing tools of different shapes and dimensions shown previously (shown in Figure 1) were used. By using differently shaped tools for burnishing under the same processing conditions, or by varying the processing parameters, it is possible to achieve different degrees of deformation of irregularities. Depending on the requirements, it is possible to achieve partial deformation of irregularities when the burnishing tool has too large a profile radius and cannot generate the specific pressures necessary to fully deform the irregularities (the valleys of the notches in the irregularities stand out on the machined surface), as well as their complete deformation when the burnishing tool exerts such high surface pressures in the burnishing zone that the irregularities are completely deformed. When analysing the burnishing process, it can be seen that material flow can lead, at sufficiently high pressures, to the accumulation of material wave uplifted on the processed surface ahead of the tool, both in the axial and circumferential directions (Figure 10).

**Figure 11:**
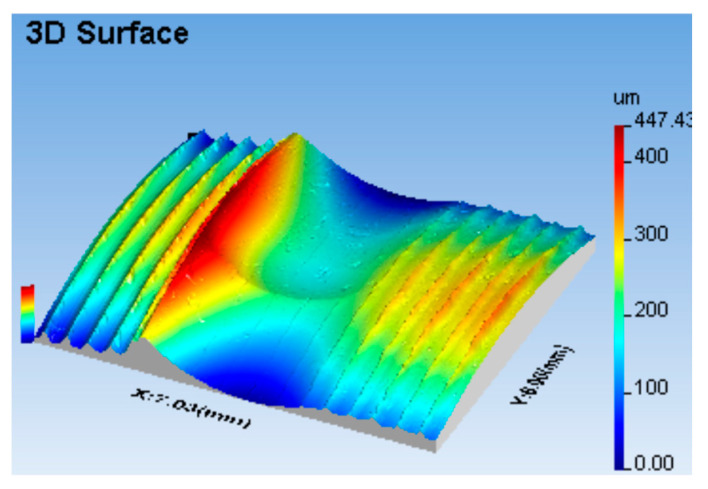
Visualisation of the material surface deformed by the burnishing tool (surface burnished with a ball indenter, R_k_ = 5 mm, F = 5 kN).

The zone of the formed irregularity had a complex, three-dimensional shape (Figure 11). It seems obvious, therefore, that a natural method of investigating such three-dimensional phenomena emerging in the area of the formed irregularity might be one that allows the analysis of a three-dimensional, stereometric image of the contact zone between the workpiece and the tool.

As part of the study, a number of analyses were carried out on the stereometric shape of the zone formed during contact between the tool and the workpiece material (C55 steel). This shape was determined by the mechanical, geometrical, and kinematic factors of the process. Important factors here are––and above all––the burnishing force, the type, shape, and dimensions of the tool and workpiece, the properties of the workpiece material, and the roughness of the starting (as-turned) surface resulting in a large extent from the feed used in upstream machining [15,16]. Measurements were carried out with the New Form Talysurf 2D/3D 120 profilographometer.

This study analysed the actual shape of the deformation zone, taking into account the kinematics of the process and the stereometry of the surface after burnishing. Consideration was given to the feasibility of using a modern technique of visualising surface stereometry to analyse the set of phenomena occurring during the formation of the tool contact zone with the workpiece material during surface treatment by burnishing. The actual shape of the area analysed, shown in Figure 11, was a slice of the lateral surface of the burnished cylinder on which the shape of the formed contact zone has been superimposed. However, this method of presenting the phenomena analysed was not convenient to use. This was because the resulting images of the forming zone were dependent on the actual diameter of the workpiece being burnished. It made it difficult to compare the results of the analyses carried out for the different burnished surfaces. It, therefore, seems appropriate to eliminate the cylindrical shape from the results obtained by digital processing of the resulting surface image.

**Figure 12:**
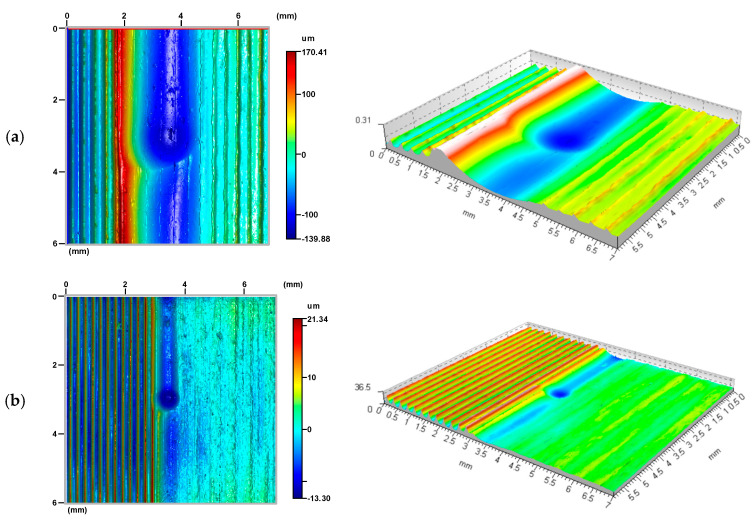
Stereometric representation of the contact zone between the burnishing tool and the burnished surface with an R = 5 mm ball indenter: (**a**) burnishing force F = 5 kN, feed values: f_turn_ = 0.410 mm/rev., f_burn_ = 0.102 mm/rev. (as-turned area: Sz = 92.4 μm, Sa = 17.5 μm; as-burnished area Sz = 72.2 μm, Sa = 9.6 μm); (**b**) burnishing force F = 0.5 kN, feed values: f_turn_ = 0.256 mm/rev., f_burn_ = 0.102 mm/rev (as-turned area: Sz = 28.5 μm, Sa = 5.5 μm; as-burnished Sz = 9.7 μm, Sa = 0.8 μm).

As part of this study, a number of analyses were carried out on the stereometric shape of the zone formed due to the contact between the tool and the machined material. During the machining process, the surface irregularities left over from the previous process were permanently deformed, and a new irregularity structure was created on the machined surface, conditioned by the kinematics of the burnishing process, with intervals between the peaks of the irregularities close to the burnishing feed rate. The zone of formed irregularity had a complex, three-dimensional shape (Figure 12).

During the burnishing process, plastic deformation occurred as a result of the burnishing tool acting on the surface irregularities, causing the material to flow in micro areas in three directions. At the start of the burnishing process, the burnishing tool only came into contact with the peaks of surface irregularities on a curve along the tool’s path. As the tool moved in the direction of the feed, the length of the contact section increased until partial or complete deformation of the irregularities occurred. There were frictional forces at the contact surface of the tool and the workpiece that impeded the deformation of irregularities in the circumferential direction. It can, therefore, be assumed that the workpiece material did not move relative to the tool surface in a direction tangential to the tool surface. Increasing pressure on the contact surfaces of the irregularities with the tool caused the material adjacent to the tool to undergo plastic flow in the direction imposed by the kinematics of the process being operated.

A stereometric image analysis of the state of the roughness of the surfaces produced by burnishing was also carried out as part of this study. As an effect of the application of surface burnishing treatment, surface irregularities left over from the previous machining were partially or completely deformed [17,18,19]. A new irregularity structure emerged, driven by the parameters of the burnishing process [20,21,22]. The resulting surface structure was the sum of the new texture obtained by the burnishing process and the residual traces of the previous machining.

**Figure 13:**
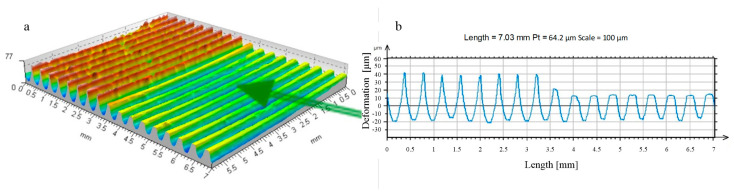
R = 13.5 mm ball-burnished surface roughness formation zone, burnishing force F = 0.5 kN, feed values: f_turn_ = 0.410 mm/rev., f_burn_ = 0.102 mm/rev. (as-turned area: Sz = 73.0 μm, Sa = 16.1 μm; as-burnished Sz = 40.7 μm, Sa = 11.4 μm) ((**a**) stereometric image, (**b**) lateral 2D deformation profile).

By analysing the processes emerging during the formation of a new texture of the surface layer by burnishing, it can be assumed that two phases of deformation of surface irregularities can be distinguished in their course. In the initial phase of their deformation, the burnishing tool in contact with the irregularities exerted high surface pressure on the peaks of irregularities. During this deformation phase, only the surface roughness peaks were deformed (Figure 13). As the pressure applied during burnishing increased, the severity of deformation of the irregularity peaks increased, leading to a reduction in the height of the irregularities remaining after the previous machining process. This may eventually lead to their complete removal [23,24]. A machining process occurred, leading to a marked improvement in the smoothness of the finished surfaces.

**Figure 14:**
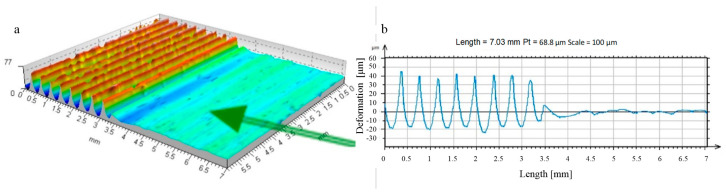
R_k_ = 5 mm ball-burnished surface roughness formation zone, burnishing force F = 0.5 kN, feed values: f_turn_ = 0.410 mm/rev., f_burn_ = 0.102 mm/rev. (as-turned area: Sz = 78.3 μm, Sa = 15.5 μm; as-burnished Sz = 13.8 μm, Sa = 1.2 μm). ((**a**) stereometric image, (**b**) lateral 2D deformation profile).

**Figure 15:**
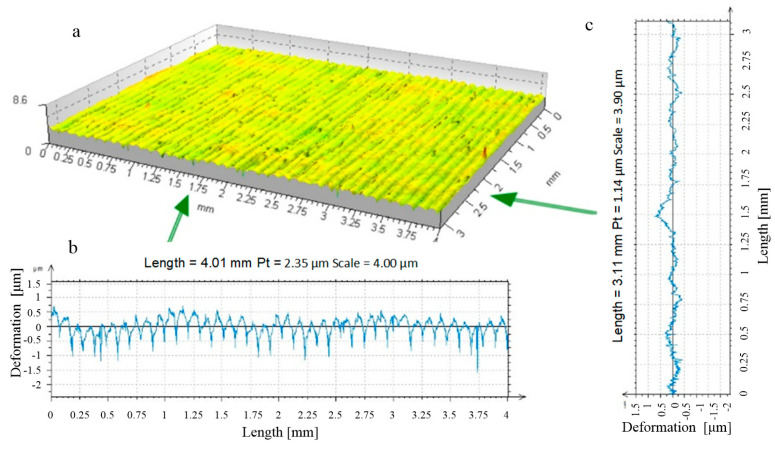
Stereometry and profile of transverse and longitudinal roughness—R = 13.5 mm ball-burnished surface, burnishing force F = 0.5 kN, feed f_burn_ = 0.102 mm/rev., (Sz = 6.1 μm, Sa = 0.3 μm). ((**a**) stereometric image, (**b**,**c**) 2D deformation profiles in the lateral and longitudinal direction).

A machining process involving only this phase of roughness deformation led to surface layers characterised by significantly improved constant and performance parameters of the surface roughness profile [25,26,27,28,29]. The improvement of the roughness parameters appears to be particularly important, as is the change in the course of the material’s curves, which leads to a noticeable improvement in the mechanical qualities of the top surface layer. This is especially important in the first period of their operation with other surfaces (in the run-in period) when the processing parameters of their roughness are decisive for the quality of the fit of the contacting surfaces [30,31,32,33]. Changes in the properties of the surface layer were not essential in this case. Plastic deformation occurred at a depth comparable to the height of the deformed roughness (Figure 14 and Figure 15). There was a noticeable reduction in the height of the irregularities (in Figure 14, as a result of the processing “Sz”, this parameter decreased from 78.3 to 13.8 μm). There was no noticeable strengthening of the material in this case, nor was there any significant change in the state of stresses residual at the depth of the surface layer. Therefore, for the surfaces machined in this way, these factors were irrelevant to the performance improvement.

When unit pressures were increased, there was a transition during machining to the next phase of roughness deformation. The material particles, under internal reactions, moved in the direction of least resistance, towards the unrestrained surfaces of the notches of the irregularities. This led to their gradual lifting (or filling up) towards the contact plane with the burnishing tool. Further on in the deformation process, when the deformation peaks (filled cavities) were already in the zone of highest pressure, slippage along the contact surface (in the axial direction) occurred, and the material flowed in the direction of the feed and also in the direction of the main rotary (circumferential) movement.

**Figure 16:**
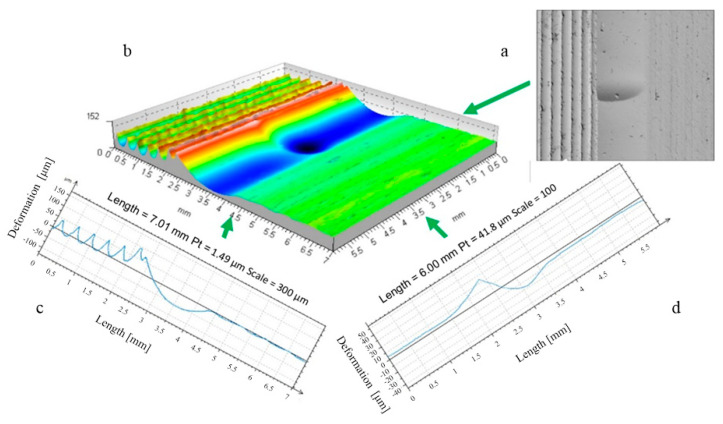
Visualisation of the material surface deformed by the burnishing tool with an R = 5 mm ball indenter, burnishing force F = 2.75 kN, feed values: f_turn_ = 0.410 mm/rev., f_burn_ = 0.102 mm/rev. (as-turned surface: Sz = 91.7 μm, Sa = 18.9 μm; as-burnished: Sz = 21.2 μm, Sa = 2.4 μm), ((**a**) top image, (**b**) stereometric image, (**c**,**d**) 2D deformation profiles in the lateral and longitudinal direction).

**Figure 17:**
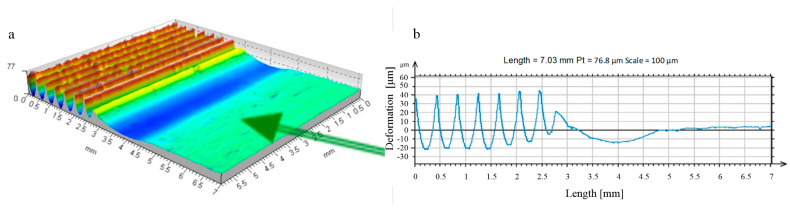
R = 13.5 mm ball-burnished surface roughness formation zone, burnishing force F = 2.75 kN, feed values: f_turn_ = 0.410 mm/rev., f_burn_ = 0.102 mm/rev. (as-turned area: Sz = 72.3 μm, Sa = 16.2 μm; as-burnished: Sz = 11.5 μm, Sa = 0.6 μm), ((**a**) stereometric image, (**b**) lateral 2D deformation profile).

**Figure 18:**
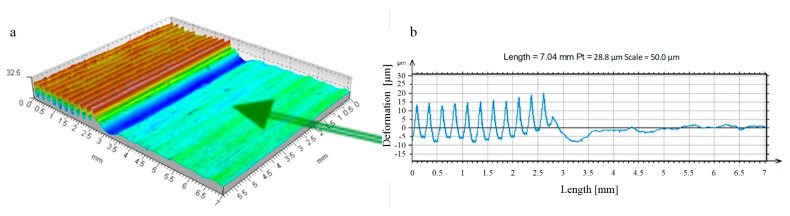
R = 5 mm ball-burnished surface roughness formation zone, burnishing force F = 0.5 kN, feed values: f_turn_ = 0.256 mm/rev., f_burn_ = 0.102 mm/rev. (as-turned area: Sz = 27.4 μm, Sa = 5.7 μm; as-burnished: Sz = 6.8 μm, Sa = 0.7 μm), ((**a**) stereometric image, (**b**) lateral 2D deformation profile).

Two waves of accumulated and deformed material formed on the surface, one oriented circumferentially and the other axially. The roughness left over from the prior machining operation was permanently deformed (Figure 16, Figure 17 and Figure 18).

**Figure 19:**
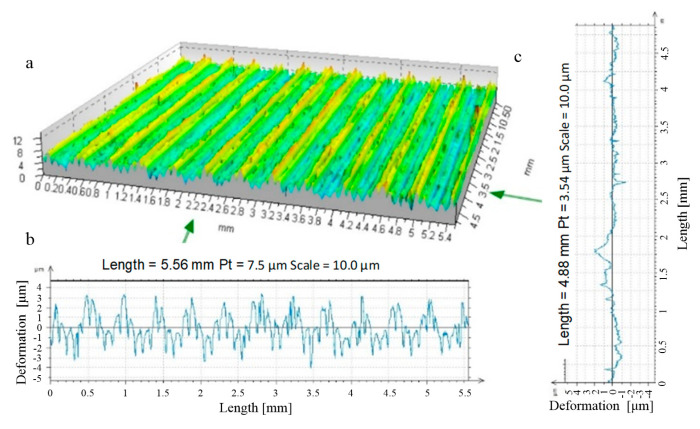
Stereometry and profile of transverse and longitudinal roughness—R_k_ = 5 mm disc-burnished surface, burnishing force F = 0.5 kN, feed f_burn_ = 0.450 mm/rev. (Sz = 13.1 μm, Sa = 1.1 μm), ((**a**) stereometric image, (**b**,**c**) 2D deformation profiles in the lateral and longitudinal direction).

During this phase of the burnishing process, only partial deformation of the irregularities could occur (Figure 19). This was the case when the burnishing tool had such a large profile radius that it was not possible to generate pressures high enough to completely deform the roughness created by burnishing. The texture of the surface formed was the result of the superposition of the texture obtained from the burnishing process, characterised by the periodicity induced by the kinematics of this process and the residual machining marks prior to burnishing. The values of the roughness parameters and the distribution of the material and ordinate distribution curves did not improve as expected. In these cases, significant changes in the physical parameters of the resulting surface layers that could lead to improvements in their performance were not expected. Thus, this type of burnishing appeared to be the least favourable for the performance achieved.

**Figure 20:**
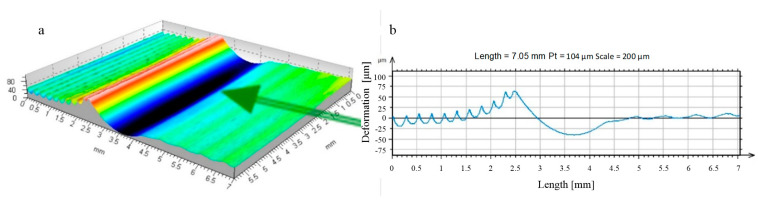
R = 5 mm ball-burnished surface roughness formation zone, burnishing force F = 2.75 kN, feed values: f_turn_ = 0.256 mm/rev., f_burn_ = 0.102 mm/rev. (as-turned area: Sz = 49.5 μm, Sa = 7.6 μm; as-burnished: Sz = 14.8 μm, Sa = 1.6 μm), ((**a**) stereometric image, (**b**) lateral 2D deformation profile).

**Figure 21:**
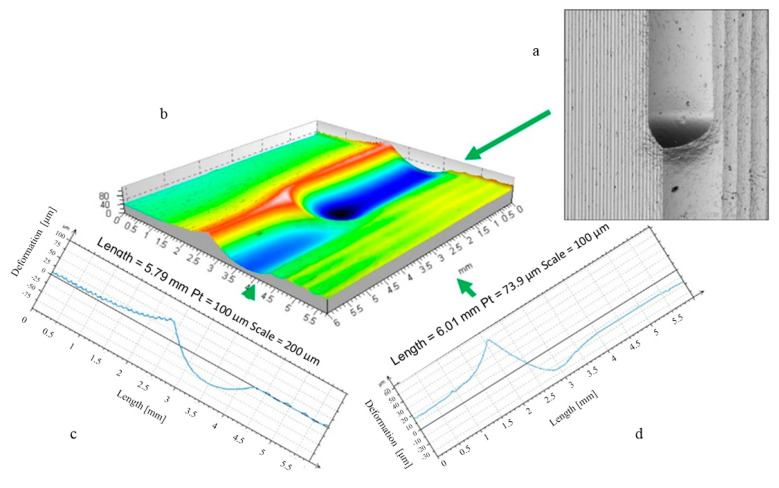
Visualisation of the material surface deformed by the burnishing tool with an R = 5 mm ball indenter, burnishing force F = 2.75 kN, feed values: f_turn_ = 0.102 mm/rev., f_burn_ = 0.410 mm/rev. (as-turned surface: Sz = 18.9 μm, Sa = 2.7 μm; as-burnished: Sz = 20.4 μm, Sa = 3.2 μm). ((**a**) top image, (**b**) stereometric image, (**c**,**d**) 2D deformation profiles in the lateral and longitudinal direction).

**Figure 22:**
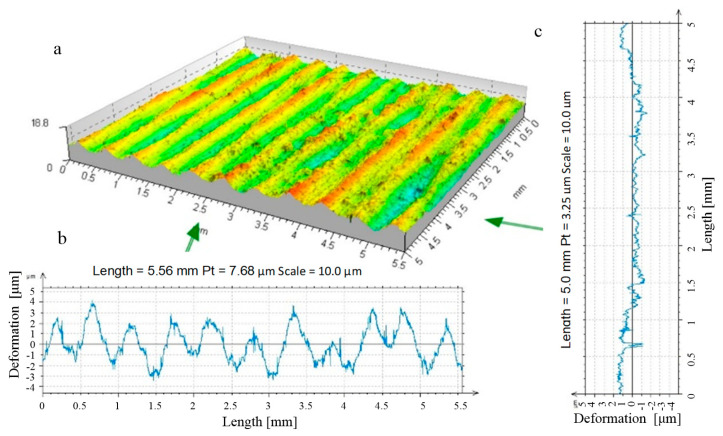
Stereometry and profile of transverse and longitudinal roughness—R = 5 mm ball-burnished surface, burnishing force F = 2.75 kN, feed f_burn_ = 0.102 mm/rev., (Sz = 14.2 μm, Sa = 1.0 μm). ((**a**) stereometric image, (**b**,**c**) 2D deformation profiles in the lateral and longitudinal direction).

It is also possible that the burnishing process results in a complete deformation of the irregularity and a new surface stereometry formed as a result of the burnishing process (Figure 20, Figure 21 and Figure 22).

It is during this phase of burnishing that the characteristic texture and cold working, manifested by an increase in hardening, was formed in the surface layer. The process of forming the surface layer in this way, which is characteristic of strengthening burnishing, is not usually characterised by a significant improvement in roughness parameters compared to pre-burnishing operations. There was also no significant improvement in the material distribution curves and the ordinate distribution of the analysed area, which is characteristic of smooth burnishing.

## 3. Results and Discussion

### 3.1. Stereometric Shape Analysis of the Deformation Zone

**Figure 23:**
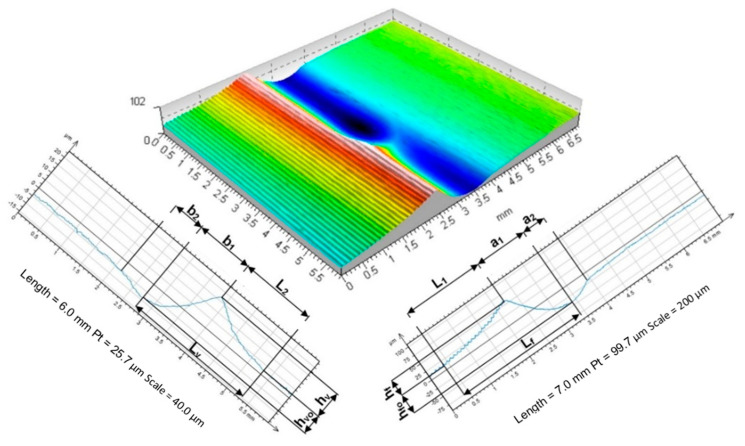
Deformation image with parameters of the deformation focus geometry.

**Figure 24:**
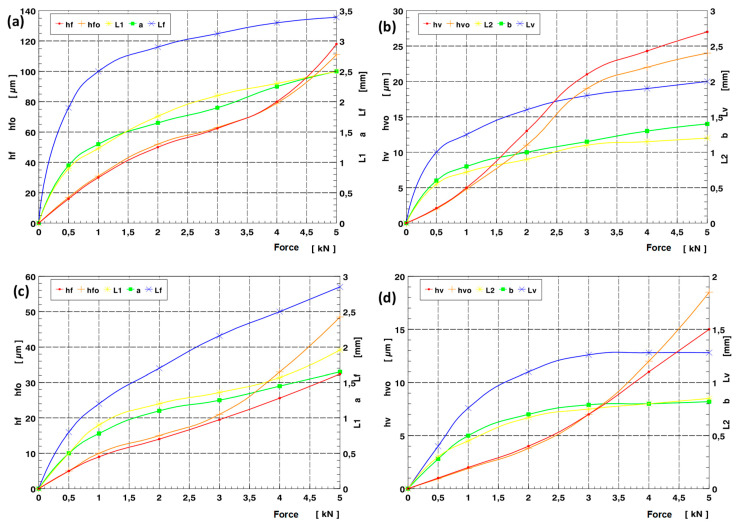
Characteristics of the effect of the burnishing force on the change of selected parameters of the deformation focus during ball burnishing (f = 0.102 mm/rev.): (**a**,**c**) determined in the axial section (h_f_, h_fo_, L_1_, a, L_f_,); (**b**,**d**) determined in the circumferential section (h_v_, h_vo_, L_2_, b, L_v_), (**a**,**b**) ball radius R = 5 mm, (**c**,**d**) ball radius R = 13.5 mm.

The digital analysis of the three-dimensional image of the acquired deformation zone described above, thanks to the digital pre-processing that allowed its levelling and the feasibility of rejecting fragments of the area of interest that were unnecessary for the analysis, also enabled a full three-dimensional analysis of the geometric parameters of the images obtained. The software (New Form Talysurf 2D/3D 120 by Taylor Hobson) used made it possible to analyse height differences in position and distance of any given area under analysis. It was possible to determine the most relevant geometrical parameters of the deformation zone. In the analyses carried out, geometric parameters describing the deformation focus determined in the axial section (in the feed plane—h_f_, h_fo_, L_1_, a = a_1_ + a_2_, L_f_,) were measured. During the analyses, the values of geometrical parameters of the deformation zone determined in the circumferential plane (plane of main motion—h_v_, h_vo_, L_2_, b = b_1_, + b_2_, L_v_) were also measured. The analysis of parameters in the circumferential section was of complementary importance. The most important geometrical parameters defining the geometrical characteristics of the deformation focus are summarised in Figure 23. The measurements completed revealed that during rolling ball pressure burnishing, the greatest impact on the increase in dimension and depth of the deformation zone formed was mainly due to the value of the tool pressure force (see charts in Figure 24).

**Figure 25:**
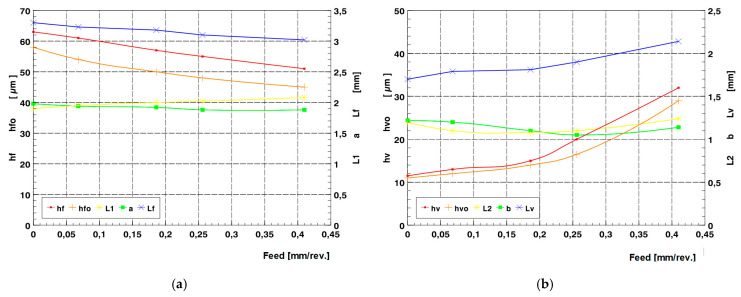
Characteristics of the effect of the burnishing feed on the change of selected deformation focus parameters during ball burnishing (R = 5 mm, F = 2.75 kN): (**a**) determined in the axial section (h_f_, h_fo_, L_1_, a, L_f_); (**b**) determined in the circumferential section (h_v_, h_vo_, L_2_, b, L_v_).

**Figure 26:**
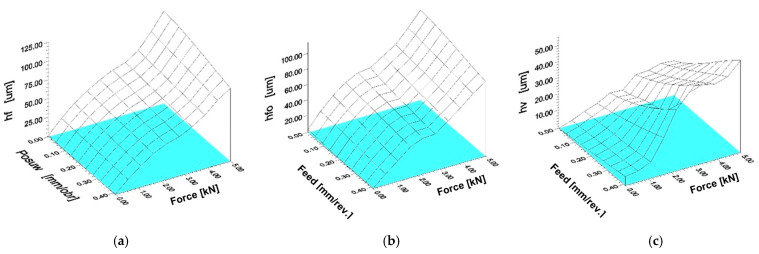
Dimensions of the axial and circumferential material wave depending on the dynamic parameters of the ball burnishing process (R = 5 mm): (**a**) h_f_(f, P), (**b**) h_fo_(f, P), (**c**) h_v_(f, P).

The course of change in geometric parameters was also dependent on the radius of the burnishing tool. For surfaces burnished with tools of larger radii (Figure 24c,d), the transition from a deformation focus of type I (characteristic of negligible unit pressures) to deformation foci of type II and III requires significantly higher burnishing forces. The impact of increasing the burnishing feed is not as conclusive. For feed rates close to zero, the course of the deformation formed was similar to the case of a circumferential groove forming on the surface of the tool path. Hence, an increase in feed rate with unchanged burnishing force did not lead to a clear increase in the deformation wave and penetration of the burnishing tool (ball indenter) (Figure 25). The analyses made it possible to determine the effect of varying the most significant processing parameters (feed and burnishing force) on changes in the geometric parameters of the deformation zone during ball pressure burnishing (Figure 26).

Similar analyses were conducted for the rolling disc burnishing process, where the dimensions of the resulting deformation zone were largely governed by the applied burnishing force. This study revealed that the final geometric characteristics of the deformation zone were influenced by factors such as the workpiece material properties, initial surface roughness, and the shape and parameters of the burnishing tool. The material was displaced both axially (along the tool’s feed direction and in the opposite direction) and circumferentially.

The analyses indicated that material flow in the axial direction, which shaped the final surface geometry, was particularly influential in determining the outcome of the process. Deformation can occur in the material region containing only the roughness peaks left by prior machining, partially smoothing them. As the burnishing force increases, a wave of displaced material begins to form, which, once exceeding a certain force, surpasses the height of these roughness peaks. This stage results in the formation of a stable deformation zone, or type III deformation, ideal for achieving the desired surface characteristics from burnishing. However, a further increase in force causes excessive deformation, potentially damaging the surface layer, and is therefore unsuitable for practical applications.

The analysis of a series of stereometric images of the deformation focus and the surfaces burnished with different machining parameters allowed the description of the deformation zone formation process. It was found that the burnishing process of interest was unstable in the initial phase. In this initial stage of deformation formation, tool penetration into the workpiece occurred. At the same time, there was an increase in the material wave in both the axial and circumferential directions. After the initial period of instability characterizing the start of the formation process of the analysed deformation zones, the parameters of the deformation focus stabilised and reached a constant value. The analyses indicate that the parameter most accurately representing the characteristics of the changes in deformation zone geometry was the deformation wave height measured in the axial section plane (*h_f_*) and the depth of the tool penetration in relation to the surface of the un-burnished material (*h_fo_*). These parameters are fairly simple to identify in industrial settings. Their measurement can also be conducted right on the machine tool with portable roughness meters.

### 3.2. Microscopic Shape Analysis of the Deformation Zone

**Figure 27:**
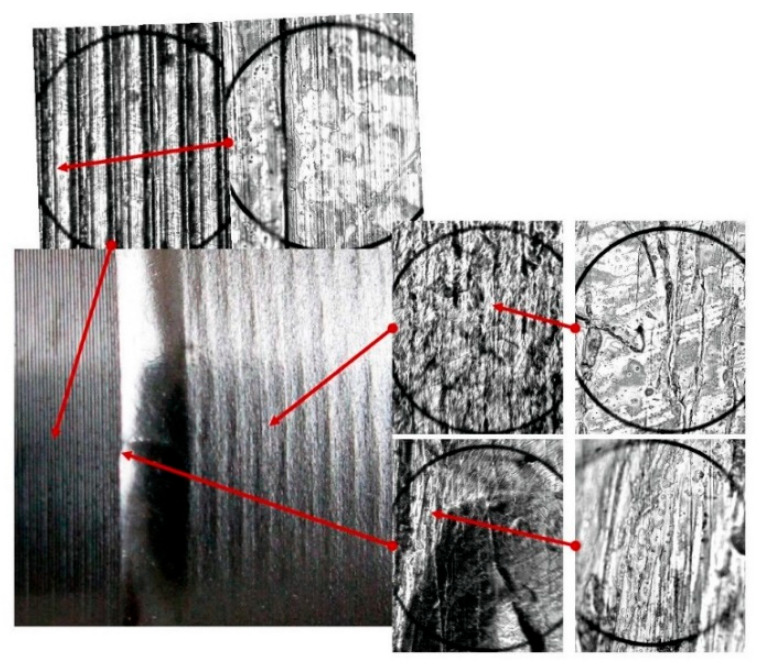
Microscopic photographs of the ball-burnished area (R = 5 mm, F = 2.75 kN, f = 0.068 mm/rev), images enlarged respectively: 30×, 100×, and 500×.

**Figure 28:**
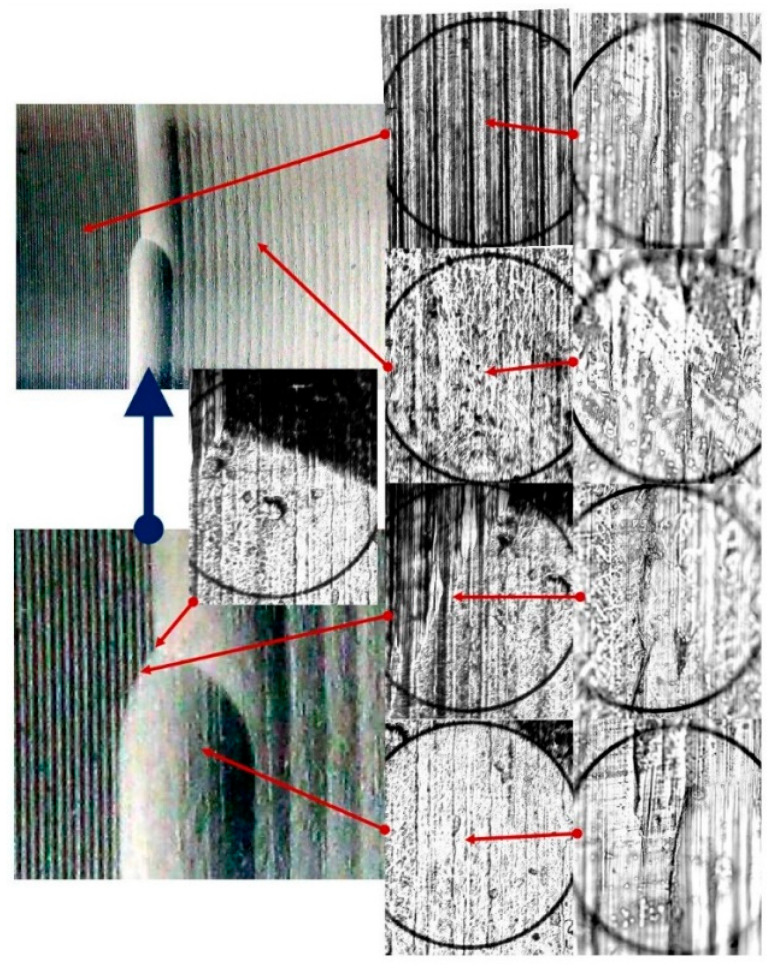
Microscopic photographs of the disc burnished area (R_k_ = 5 mm, F = 2.75 kN, f = 0.41 mm/rev), images enlarged respectively: 30×, 100×, and 500×.

The study of the stereometry of the deformation zone was complemented by an analysis of microscopic photographs revealing the changes occurring on the surface of the workpiece. The capabilities of the laboratory digital microscope—the VMHT MOT microhardness tester—were used. The images presented allowed analysis of the material structure in selected areas of the deformation zone (Figure 27 and Figure 28).

The distinctive texture created by the turning process was substituted with the texture produced by the plastic deformation caused by the movement of the burnishing tool (Figure 27). Captured at various magnifications, the microscopic images displayed the material composition of both the turned zone and the area formed by the movement of the burnishing tool. A distinct material boundary is observable in the machining initiation area (deformation zone boundary). This was the region of significant plastic deformation that initiated the formation process of this area. A new surface texture emerged on the machined surface due to the deformation.

The deformation pattern observed during the rolling disc burnishing process (Figure 28) exhibited a comparable trend. As a result of the high feed rate utilized during the burnishing process, the surface irregularities created from the kinematic reproduction of the burnishing disc also became apparent. Photos captured at various magnifications (Figure 28) showed alterations in the composition of the surface layer of the material.

The observation of phenomena occurring in the deformation focus was particularly important from the point of view of the process under analysis. In the deformation area, the roughness left after turning was re-formed. The procedure began at the limit created by the kinematic replication of the tool shape. The summits of anomalies were plastically shaped. The entire area was altering its nature due to the ongoing deformation. Instead of the longitudinally aligned marks of the cutting-edge transition typical of the turning process, along with its serrations, the material’s texture emerged on the surface due to plastic deformation. Within the deformation zone beneath the burnishing tool, the deformation impressions of the plastically deformed material were observed, with gaps arising from the roughness left by prior machining processes (specifically turning). These marks, due to deformation happening in the region where the tool exited the machining zone, were no longer apparent in the area of the surface modified by burnishing. In this region, there had already been total plastic deformation and re-shaping of the pre-burnishing inconsistencies. A new texture of the top layer of the material was created.

**Figure 29:**
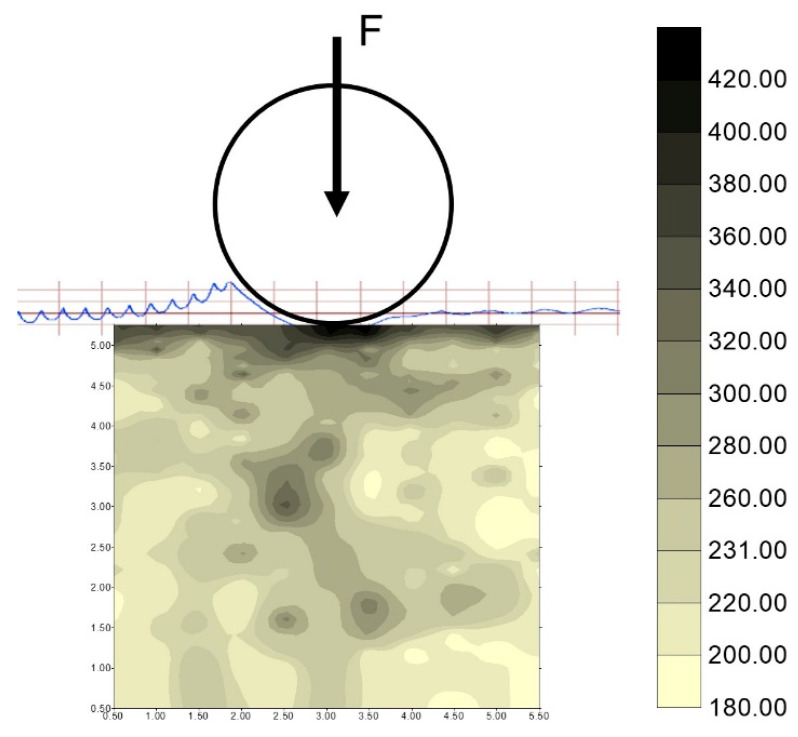
Microhardness distribution in the zone of formed irregularity for the R_k_ = 5 mm ball-burnished surface, burnishing force F = 5 kN.

The improvement in the performance of the modified surface layer was attributed to the improved state of its physical parameters [34,35,36]. In these cases, there was a significant strengthening of the outer layer occurring at depths of up to several millimetres [37,38]. What is important here is not only the value of the microhardness obtained, but also its distribution at the depth of the surface layer as a result of the deformation occurring within the deformed area (Figure 29). Strengthening had the beneficial effect of improving the material’s resistance to wear and tear under dry friction conditions [39,40,41]. Previous research indicates that the introduction of substantial compressive stresses into the surface layer is also crucial for enhancing the performance of outer surfaces [42]. The values and distribution of the resulting stresses stem from a series of phenomena that take place during the process of deformation while burnishing.

## 4. Conclusions

This research investigated the effect of the pressure burnishing process on the formation of the surface layer. As a result of the conducted comprehensive analyses, many beneficial effects were demonstrated in terms of improving the mechanical properties of the surface layer undergoing processing. The significant benefits are listed in the following points:

(1) The geometric characteristics of the deformation zone are shaped by factors such as the properties of the machined material, the initial surface roughness, and the burnishing tool’s shape and processing parameters. During burnishing, the deformed material shifts both in the axial direction (aligned with and opposite to the tool feed) and circumferentially.
(2) As demonstrated, axial material flow is critical for achieving the desired final surface structure in the burnishing process. The deformation primarily affects material regions containing the peaks of roughness left by previous machining, leading to only partial smoothing of these peaks. As the burnishing force increases, a wave of displaced material forms and grows, eventually exceeding the height of the initial roughness peaks when a specific force threshold is crossed. Under these conditions, a stable deformation zone (or type III deformation) forms, which is ideal for obtaining the optimal surface properties through burnishing.
(3) The undertaken analysis of a series of stereometry images of the deformation focus and the machined burnished surfaces with different processing parameters allowed for the description of the process of shaping the deformation zone. It was observed that the analysed burnishing process was unstable in the initial phase. In this initial stage of forming the deformation, the tool was penetrating the machined material. At the same time, the material wave was growing in both the axial and circumferential directions. After the initial period of instability, which characterised the beginning of the process of shaping the analysed deformation zones, the parameters of the deformation focus stabilised and reached a constant value.
(4) The practical application of measurement stations for simulation analysis of specific stereometry parameters and deformation zone shapes in burnished surfaces significantly enhances the quantity and relevance of information gathered post-processing. This approach allows for precise determination of the burnishing tool’s contact area with the processed material, information that was previously unattainable, thus enabling accurate measurement of contact stress values within the contact zone. It also facilitates the monitoring of shape changes within the deformation zone in both axial and circumferential cross-sections.
(5) Analyses indicate that the most representative parameters describing changes in the deformation zone geometry are the deformation wave height (h_f_), measured in the axial cross-section, and the depth of tool penetration relative to the unprocessed material surface (h_fo_).

The outcomes of the research provide a starting point for further analyses aimed at linking the description of the deformation zone forming process with the resulting changes in the microhardness distribution and stress state in the contact zone. This would facilitate the optimal selection of technological processing parameters, enabling the production of burnished surface layers with significantly improved usage properties.

## Data Availability

The original contributions presented in the study are included in the article, further inquiries can be directed to the corresponding author.
